# Ten years of experience of biologics in juvenile idiopathic arthritis: focus for the reasons of withdrawals

**DOI:** 10.1186/1546-0096-12-S1-P140

**Published:** 2014-09-17

**Authors:** Svetlana V Arsenyeva, IP Nikishina, MI Kaleda, LG Medyntceva, SR Rodionovskaya, DL Alexeev, ES Fedorov

**Affiliations:** 1 Paediatric, "Nasonova research Institute of Rheumatology", Moscow, Russian Federation

## Introduction

Biologics are often used in therapy of DMARDs resistant JIA. The important problem is discontinuation of Biologics therapy due to different reasons.

## Objectives

Evaluation of 10 years of the experience of Biologics in children, suffering from juvenile idiopathic arthritis (JIA) in single center focused for reasons of withdrawal.

## Methods

The analysis includes data about 435 patients with JIA who have been getting Biologics in 2005 - 2014. The average age of patients is 10,5 years (from 1,5 to 18 years). Disease duration is 7 yrs avg (from 3 to 14 years). Clinical characteristics: soJIA - 70 (17%), JIA with polyarthicular course (poly) - 257 (61%), JIA oligo – 38 (9%), 51 (12%) patients suffered from active uveitis.

## Results

During the observation Biologics were withdrawn in 123 cases (98 patients). For some patient we changed Biologics several times: in 21 cases - two times (21% of patients), in 2 cases (2%) - thrice. Distribution of reasons for the Biologics discontinuation is presented in Table 1 below:

**Table 1:** 

	All biologics	Infliximab	Abatacept	Tocilizumab	Adalimumab	Etanercept
**Withdrawal reasons**						

Adverse events	40	25	3	5	2	4

Inefficacy	51	21	16	1	4	5

Other	32	14	1	2	13	2

**Therapy duration before withdrawal**						

Half a year	4	2	0	2	0	0

One year	36	16	7	2	7	3

Two years	17	7	4	1	0	3

Three years and more	17	11	2	0	2	1

Infliximab was withdrawn more often due to adverse events (infusion reactions), at the beginning of the treatment or several years later. The other reason is secondary inefficiency after 2-5 years of application. Abatacept was cancelled more often for the reason of inefficiency, adverse events were observed rarely. Etanercept was withdrawn in some cases because of uveitis de novo. Adalimumab was withdrawn basically due to organization problem.

Favorable choice of Biologics is changed from 2005 till now. For new initiation we used different biologics in different time. Infliximab was administered in the past (from 2005 to 2012), maximum (14 patients) in 2011. Administration number decrees nowadays (3 in 2013, 1 in 2014). Adalimumab was not commonly administrated before 2011, from 2012 administration count increase from 10-11 per year to 39. Using of Etanercept was increased from 2010 (20-35) and achieved maximum in 2013 (49 patients). Usually we prescribe tocilizumab in systemic JIA and do not change in most cases.

## Conclusion

Availability of Biologics therapy was increased during last ten years in Russia. That has improved survival of therapy and has given opportunity of using Biologics with good safety profile.

## Disclosure of interest

None declared

